# Effects of Exercise and Omega-3-Supplemented, High-Protein Diet on Inflammatory Markers in Serum, on Gene Expression Levels in PBMC, and after Ex Vivo Whole-Blood LPS Stimulation in Old Adults

**DOI:** 10.3390/ijms24020928

**Published:** 2023-01-04

**Authors:** Ulrike Haß, Sarah Heider, Bastian Kochlik, Catrin Herpich, Olga Pivovarova-Ramich, Kristina Norman

**Affiliations:** 1 Department of Nutrition and Gerontology, German Institute of Human Nutrition Potsdam-Rehbruecke, 14558 Nuthetal, Germany; 2 Institute of Nutritional Science, University of Potsdam, 14558 Nuthetal, Germany; 3 Research Group Molecular Nutritional Medicine, German Institute of Human Nutrition Potsdam-Rehbruecke, 14558 Nuthetal, Germany; 4 Department of Geriatrics and Medical Gerontology, Charité—Universitätsmedizin Berlin, Corporate Member of Freie Universität Berlin and Humboldt-Universität zu Berlin, 13347 Berlin, Germany; 5 Department of Endocrinology, Diabetes and Nutrition, Charité—Universitätsmedizin Berlin, Corporate Member of Freie Universität Berlin, Humboldt-Universität zu Berlin, and Berlin Institute of Health, 10117 Berlin, Germany; 6 German Center for Diabetes Research (DZD), 85764 München-Neuherberg, Germany; 7 German Centre for Cardiovascular Research (DZHK), Partner Site Berlin, 10785 Berlin, Germany

**Keywords:** inflammaging, immunosenescence, cytokines, gene expression, peripheral blood mononuclear cells, lipopolysaccharide stimulation, omega-3 fatty acids, high-protein diet, whole-body vibration exercise

## Abstract

Inflammaging is related to cell senescence and reflects an erratic immune system, which promotes age-associated diseases. Exercise and nutrition, particularly omega-3 fatty acids, are able to affect inflammation. Therefore, we examined the effects of an 8-week exercise and dietary intervention on the inflammatory response in community-dwelling old adults. All participants received weekly vibration and home-based resistance exercise. Furthermore, participants were randomized to either a control, high-protein (1.2–1.5 g/kg), or high-protein, omega-3-enriched (2.2 g/day) diet. Before and after treatment, inflammatory markers in fasting serum and after whole-blood ex vivo lipopolysaccharide (LPS) stimulation were assessed. Gene expression levels of inflammatory markers were quantified in peripheral blood mononuclear cells (PBMC). Sixty-one participants (age: 70.6 ± 4.7 years; 47% men) completed the study. According to generalized linear mixed models, a high-protein, omega-3-enriched diet decreased circulating anti-inflammatory interleukin (IL-) 10 and IL-1 receptor antagonist (IL-1RA). Sex-stratified analyses showed also significantly reduced pro-inflammatory markers in men with a high-protein, omega-3-enriched diet. Gene expression of IL-1RA was significantly reduced after both protein-enriched diets compared with controls. In comparison to a high-protein diet, exercise alone showed lower LPS-induced release of c-c motif chemokine ligand-2 (CCL-2), which tended to be more pronounced in men compared with women. Eight weeks of a high-protein, omega-3-enriched diet combined with exercise decreased circulating anti-inflammatory markers, and pro-inflammatory markers in men. A high-protein diet attenuated anti-inflammatory markers on gene expression level in PBMC. Exercise alone resulted in a lower pro-inflammatory response to LPS-exposure in whole-blood cultures.

## 1. Introduction

A persistent low-grade inflammation in higher age, also known as inflammaging, alters cell function and fuels age-related diseases such as cardio-metabolic diseases as well as musculoskeletal disorders, such as sarcopenia [1]. For instance, inflammation-driven macrophage infiltration into arterial walls and in pancreas causes atherosclerotic plaque formation and β-cell apoptosis, which in turn leads to atherosclerosis and insulin resistance, respectively [2]. Furthermore, it is known that chronic inflammation inhibits growth factors and activates proteolytic pathways, resulting in loss of skeletal muscle [3]. Underlying mechanisms of inflammaging are complex and include increasing cellular senescence and accumulation of endogenous cell debris (damage-associated molecular patterns) [1]. Furthermore, immune senescence and senescent cells, which particularly accumulate in visceral adipose tissue, stimulate an innate inflammatory response (senescence-associated secretory phenotype) [4]. Moreover, old adults exhibit less naïve B cells and regulatory T cells, while memory regulatory T cells show increased numbers. This decrease in immune cell diversity increases failure to self-tolerance, resulting in increased inflammation, impaired host defense against pathogens, and an elevated risk of autoimmunity in old adults [1,4]. In particular, non-dividing, aged B cells, which are present in inflammatory processes, are able to secrete cytokines and are involved in antibody production associated with autoimmunity [4].

The term “inflamm-aging” has recently been extended to “inflamm-inactivity”, since it is recognized that a sedentary lifestyle causes a relevant proportion of inflammatory processes [5], which are reversible with regular exercise even in higher age, as different meta-analyses have shown [6,7]. Furthermore, a lower dietary inflammatory burden has been associated with lower systemic inflammation in old adults [8]. In particular, omega-3 polyunsaturated fatty acids (omega-3) have direct and indirect anti-inflammatory properties due to altered cell membrane phospholipid fatty acid composition with modulation of pro- and anti-inflammatory transcription factor activity, resulting in reduced inflammatory gene expressions [9,10,11]. A recent meta-analysis affirmed the beneficial effects of omega-3 supplementation on common inflammatory markers such as c-reactive protein (CRP), interleukin (IL-) 6, and tumor necrosis factor (TNF-) α under various health conditions [10]. Although still under debate, evidence further suggests that proteins in particular from dairy/whey or soy have anti-inflammatory potential [12,13,14,15]. We recently performed an intervention trial to assess the physical and biochemical effects of whole-body vibration training and home-based exercise in combination with a high-protein, omega-3-enriched diet in community-dwelling old adults [16]. Hereby, we observed significant alterations of several circulating inflammatory markers with additional omega-3 supplementation [16]. Therefore, the primary aim of this sub-analysis was the detailed investigation of the impact of an omega-3-supplemented, high-protein diet in combination with exercise on inflammatory status in old adults using analyses of inflammatory markers in serum, on gene expression levels in peripheral blood mononuclear cells (PBMC), and in lipopolysaccharide (LPS)-stimulated whole-blood cultures. Since we observed sex-specific differences in our previous analyses [16] and it is recommended to consider the impact of sex on inflammation even in higher age [17], we also examined sex-related inflammatory responses in sub-group analyses.

## 2. Results

### 2.1. Baseline Characteristics

In total, 61 old adults (70.6 ± 4.7 years; 53% women) were included in the final analysis. The three groups, consisting of either a control, a high-protein (protein), or a high-protein, omega-3-enriched diet (protein + omega-3), showed similar baseline characteristics such as age, sex distribution, body composition (Table 1) as well as physical fitness and dietary intakes (see Table S1 as published in [16]). Baseline values of inflammatory markers were also comparable between groups, aside from serum IL-1RA (Table 1).

### 2.2. Adherence to Interventions and Changes in Body Composition

With regard to our exercise intervention, the participants started with overall comparable physical fitness and showed similar adherence to exercise protocols regarding vibration training as well as home-based exercises (see Table S1 as published in [16]).

The high adherence rate of omega-3 supplementation resulted in a significantly increased omega-3 plasma index only in the protein + omega-3 group (see Table 1 as published in [16]). Moreover, high compliance rates of whey supplementation in the protein and protein + omega-3 group resulted in significantly increased protein intakes in both groups (see Table S1 as published in [16]). Due to the higher protein intakes, fat intakes significantly decreased in both protein-enriched groups compared with control (see Table S1 as published in [16]). However, further relevant dietary intakes, such as caloric and carbohydrate intake, did not change significantly between groups (see Table S1 as published in [16]).

Although the body mass index (BMI) significantly increased only within the protein + omega-3 group (+0.26 ± 0.43 kg/m^2^, *p =* 0.011), changes in fat mass index (FMI) were comparable between the control (+0.15 ± 0.73 kg/m^2^), the protein (−0.24 ± 0.82 kg/m^2^), and the protein + omega-3 (−0.18 ± 0.82 kg/m^2^) group (overall *p =* 0.257).

### 2.3. Omega-3 Supplementation Decreased Circulating Inflammatory Markers

**Figure 1:**
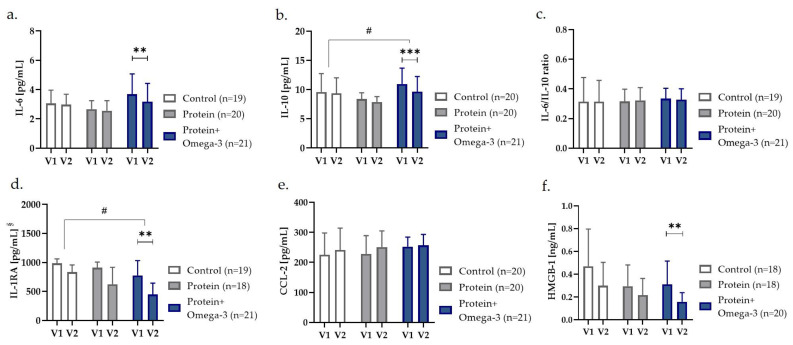
Fasting serum concentrations of (**a**) IL-6, (**b**) IL-10, (**c**) IL-6/IL-10 ratio, (**d**) IL-1RA, (**e**) CCL-2, and (**f**) HMGB-1 presented as estimated mean with 95% confidence interval before (V1) and after (V2) eight weeks of control, high-protein (protein) or high-protein, omega-3-enriched (protein + omega-3) diet. ** *p* < 0.01, *** *p* < 0.001 obtained from within-group comparisons with either paired *t*-test or Wilcoxon rank test, and ^# ^*p* < 0.05 indicating group × time-interaction effects between groups in generalized linear mixed models with random effects on subjects, adjusted for age, sex, and fat mass index, ^§^ and due to significantly different cases at baseline, also adjusted for baseline values. CCL-2 c-c motif chemokine ligand-2; HMGB-1 high-mobility group box-1; IL interleukin, RA receptor antagonist.

To investigate intervention effects on inflammatory markers in old adults, we first assessed the circulating levels of IL-6, IL-10, IL-1 receptor antagonist (IL-1RA), c-c motif chemokine ligand-2 (CCL-2), and high-mobility group box-1 (HMGB-1) in fasting serum samples. According to unadjusted within-group comparisons before and after treatment, serum concentrations of IL-6 (*p* = 0.005), IL-10 (*p* < 0.001), IL-1RA (*p* = 0.001), and HMGB-1 (*p* = 0.001) significantly decreased in the protein + omega-3 group (Figure 1). Fully adjusted mixed models confirmed significant decreased IL-10 (*p* = 0.041) and IL-1RA (*p* = 0.016) serum concentrations in the protein + omega-3 group compared with control (Figure 1).

Subsequent sex-stratified analyses indicated that additional omega-3 supplementation led to significantly decreased IL-10 in both women (*p* = 0.021) and men (*p* = 0.004) (Figure S1), while in men, also significantly decreases in IL-6 (*p* = 0.005), CCL-2 (*p* = 0.010), and HMGB-1 (*p* = 0.049) were found compared with control (Figure S1). Moreover, compared with the protein group, additional omega-3 supplementation in men resulted in significantly reduced CCL-2 (*p* = 0.004) and HMGB-1 (*p* = 0.001), and also showed a trend towards decreased IL-6 (*p* = 0.095) (Figure S1). Furthermore, a lower IL-6/IL-10 ratio (*p* = 0.053) was observed in the male protein + omega-3 group compared with control (Figure S1), and IL-1RA concentrations tended to decrease in the female (*p* = 0.093) as well as in the male (*p* = 0.099) protein + omega-3 group compared with control (Figure S1). Comparison between sexes revealed that the changes in IL-10 (*p* = 0.028) and CCL-2 (*p* = 0.011) were significantly different between women and men, while the change in IL-6 showed a trend (*p* = 0.075) (Figure S1).

### 2.4. Gene Expression Levels of Inflammatory Markers in PBMC Are Reduced with Both Protein-Enriched Dietary Interventions

**Figure 2:**
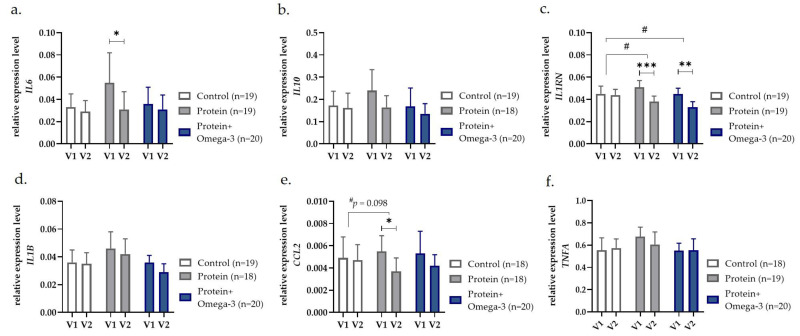
Gene expression levels analyzed in peripheral blood mononuclear cells of (**a**) *IL6*, (**b**) *IL10*, (**c**) *IL1RN*, (**d**) *IL1B*, (**e**) *CCL2*, and (**f**) *TNFA* presented as estimated mean with 95% confidence interval before (V1) and after (V2) eight weeks of control, high-protein (protein) or high-protein, omega-3-enriched (protein + omega-3) diet. * *p* < 0.05, ** *p* < 0.01, *** *p* < 0.001 obtained from within-group comparisons with either paired *t*-test or Wilcoxon rank test, and ^# ^*p* < 0.05 indicating group × time-interaction effects between groups in generalized linear mixed models with random effects on subjects, adjusted for age, sex, and fat mass index. CCL2 c-c motif chemokine ligand-2; IL interleukin, B beta, RN receptor antagonist; TNFA tumor necrosis factor-α.

To better understand intervention effects on immune cells in old adults, we analyzed expression levels of genes coding cytokines/chemokines in isolated PBMC. Within-group comparison showed significant reductions in gene expression levels of IL-6 (*IL6*; *p* = 0.030) and CCL-2 (*CCL2*; *p* = 0.011) exclusively in the protein group (Figure 2). Furthermore, IL-1RA (*IL1RN*) expression levels significantly decreased in the protein (*p* < 0.001) as well as in the protein + omega-3 group (*p* = 0.001) (Figure 2). Adjusted mixed models confirmed a significant reduction in *IL1RN* after eight weeks of a protein (*p* = 0.013) as well as a protein + omega-3 diet (*p* = 0.018) compared with control (Figure 2). In addition, the protein group showed a trend towards reduced gene expression levels of *CCL2* compared with control (*p* = 0.098) (Figure 2).

In women, sex-stratified analyses indicated in the protein as well as the protein + omega-3 group significant reductions on gene expression levels of *IL6* (*p* = 0.016 and *p* = 0.004) and *IL1RN* (*p* = 0.010 and *p* = 0.004) (Figure S2). In men, the protein group showed significantly decreases in gene expression levels of *CCL2* compared with control (*p* = 0.019) (Figure S2), and a trend towards lower gene expression levels of *CCL2* (*p* = 0.067) and TNF-α (*TNFA*; *p* = 0.090) compared with the protein + omega-3 group (Figure S2). Although no statistically significant changes in IL-1β (*IL1B*) gene expression levels were found within sexes, the male protein + omega-3 group showed a significantly greater decline in expression levels compared with the female protein + omega-3 group (*p* = 0.025) (Figure S2). However, no further sex-specific response was observed for gene expression levels.

### 2.5. Reduction in Ex Vivo LPS-Stimulated Cytokine/Chemokine Release after Exercise and Dietary Interventions

**Figure 3:**
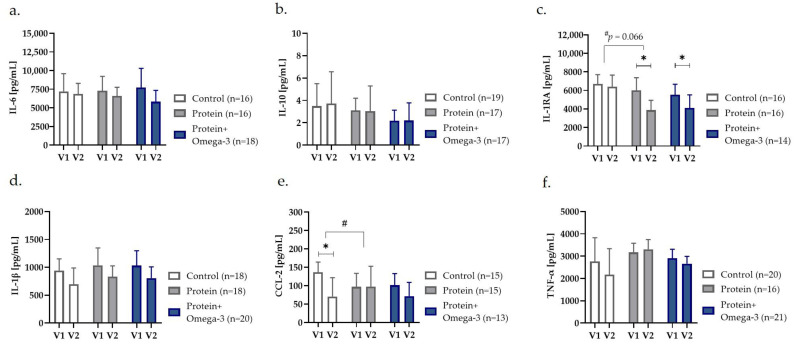
Ex vivo whole-blood LPS-induced concentrations of (**a**) IL-6, (**b**) IL-10, (**c**) IL-1RA, (**d**) IL-1β, (**e**) CCL-2, and (**f**) TNF-α presented as estimated mean with 95% confidence interval before (V1) and after (V2) eight weeks of control, high-protein (protein) or high-protein, omega-3-enriched (protein + omega-3) diet. * *p* < 0.05 obtained from within-group comparisons with either paired *t*-test or Wilcoxon rank test, and ^#^ *p* < 0.05 indicating group × time-interaction effects between groups in generalized linear mixed models with random effects on subjects, adjusted for age, sex, and fat mass index. CCL-2 c-c motif chemokine ligand-2; IL interleukin, RA receptor antagonist; TNF-α tumor necrosis factor-α.

To investigate how exercise and dietary interventions affect the immune cell capacity of old adults, we evaluated the LPS-stimulated secretion levels of cytokines/chemokines in whole-blood cultures. Unadjusted within-group comparison indicated a significantly diminished LPS-induced production of IL-1RA in the protein group (*p* = 0.020) as well as protein + omega-3 group (*p* = 0.030) (Figure 3). However, only the protein group showed a trend towards decreased IL-1RA concentrations compared with control (*p* = 0.066) in adjusted mixed models (Figure 3).

Furthermore, within-group comparisons depicted a significant lower LPS-induced release of CCL-2 after eight weeks exclusively in the control group (*p* = 0.046) (Figure 3). Mixed model analyses confirmed significantly differences of the intervention effects in the control vs. protein group (*p* = 0.046) (Figure 3).

In women, a significant reduced LPS-induced release of IL-1RA in the protein group (*p* = 0.044) and a trend towards reduced IL-1β release in the protein + omega-3 group (*p* = 0.051) was observed compared with control (Figure S3). In men, CCL-2 release significantly reduced in the control group compared with the protein group (*p* = 0.004) and tended also to be lower in the protein + omega-3 compared with the protein group (*p* = 0.096) (Figure S3). Moreover, the male protein + omega-3 group showed a trend towards greater decline in LPS-induced TNF-α release compared with the protein group (*p* = 0.064) (Figure S3). Furthermore, direct comparison of changes between women and men showed a trend towards a stronger decline in CCL-2 (*p* = 0.072) as well as IL-1β release (*p* = 0.080) in the male compared with the female control group (Figure S3).

## 3. Discussion

To the best of our knowledge, the present sub-analysis is the first to investigate the impact of a whey protein-enriched, omega-3-supplemented diet in combination with whole-body vibration training and home-based exercise on the inflammatory status in community-dwelling old adults, using the combination of analyses in serum, in PBMC, and in ex vivo LPS-stimulated whole-blood cultures. A high-protein, omega-3-enriched diet (for 8 weeks) resulted in significantly decreased circulating anti-inflammatory IL-10 and IL-1RA. Moreover, circulating pro-inflammatory IL-6, CCL-2, and HMGB-1 decreased with omega-3 supplementation in men. Gene expression levels of *IL1RN* were significantly reduced with both a high-protein and high-protein, omega-3-enriched diet. According to mixed models, exercise alone compared with exercise combined with a high-protein diet showed a diminished LPS-induced release of CCL-2 in whole-blood cultures, which is likely attributable to men.

### 3.1. Isolated Effects of Exercise Intervention on LPS-Induced Chemokine Release

Details regarding the dietary compliance rates and exercise adherence of our participants were recently published [16]. In brief, the participants were comparably compliant to training protocols regarding vibration training and home-based exercises [16]. Aside from significantly lower LPS-induced CCL-2 release in whole-blood cultures, we observed no further significant changes in inflammatory markers on protein or gene expression levels in our control group, receiving only exercise. This is unexpected, since it has been reported that exercise in general is improving the inflammatory response in old adults [7]. However, our results are in line with another study, which observed no changes in IL-6, IL-10, IL-1β, and TNF-α on protein and gene expression levels after nine weeks of vibration training (3 days/week) in community-dwelling old adults [18].

Another study performing 12 weeks of moderate strength exercises with 45 healthy old women also observed no changes in IL-2, IL-6, IL-10, TNF-α, and interferon-γ, produced by T lymphocytes [19].

Home-based resistance exercise programs are effective in counteracting sedentariness [20], but are relatively mild-to-moderate interventions compared with progressive, supervised strength training. Therefore, our combined exercise protocol may have been not intense enough to gain significant changes on the immune response in healthy old adults.

### 3.2. Decreased Inflammatory Markers in Serum after High-Protein, Omega-3 Enriched Diet

Although there is a lack of knowledge about interaction effects between exercise and omega-3 supplementation, particularly in old adults, it is assumed that omega-3 acts complementary to exercise in immunomodulatory and anti-inflammatory processes [21]. This is in line with our observation on circulating inflammatory markers. At least in men, exercise in combination with a high-protein, omega-3-enriched diet led to significant reduced concentrations of IL-6, CCL-2, and HMGB-1 (Figure S1). The additional effects of omega-3 were moreover confirmed by direct comparisons between the protein and the protein + omega-3 group (Figure S1). We also found a reduction in the anti-inflammatory IL-10 and IL-1RA, which might be interpreted as a compensatory effect to the decreasing pro-inflammatory concentrations. However, there is a lack of studies which investigate the combined effect of training and diet on pro-inflammatory and anti-inflammatory markers in healthy old adults.

### 3.3. Gene Expression Levels of Inflammatory Markers Reduced with Protein-Enriched Diets

We observed significantly lower gene expression levels of *IL6* and *IL1RN* with both protein-enriched diets in women. Furthermore, *CCL2* expression was significantly lower in men after the high-protein intervention. Literature on the influence of dietary protein on inflammation is controversial and may depend on the protein source and amino acid composition. In 37 patients (64 ± 6 years) with type 2 diabetes and non-alcoholic fatty liver disease, six weeks of an animal- or plant-based high-protein diet resulted in equally reduced inflammation [13,22]. Indeed, evidence suggests that proteins particularly from dairy/whey or soy have also anti-inflammatory potential [12,23]. Whey consists of different proteins, including β-lactoglobulin, α-lactalbumin, glycomacropeptide, and lactoferrin, possessing anti-inflammatory and immune-regulatory properties. The anti-inflammatory effect of whey has recently been shown after 13 weeks of supplementation in old adults with sarcopenia [15] as well as after even 3 weeks in 42 patients with acute stroke [14].

### 3.4. Attenuated or no Change in LPS-Induced Immune Response with Dietary Interventions

In our study, we used the ex vivo whole-blood assay which is a promising method to assess the cytokine release and cell activation in response to dietary or exercise intervention. It has been demonstrated that old compared with young adults show an attenuated pro-inflammatory response to LPS exposure, possibly reflecting an impaired host defense against pathogens [24]. A study performed in healthy old women, found an increased production of concanavalin A- and LPS-induced cytokine release from lymphocytes after 12 weeks of strength exercise and omega-3 supplementation, which was interpreted as improved immune system and functioning of neutrophils [19]. In contrast, we observed a trend towards lower LPS-induced concentrations of IL-1β in the female protein + omega-3 group, and lower CCL-2 and TNF-α release in the male protein + omega-3 group (Figure S3). Interestingly, in patients with Alzheimer’s disease, significantly decreased LPS-induced release of IL-6 and IL-1β in PBMC after six months of omega-3 supplementation (2.3 g/day) was reported [25]. However, in 46 healthy (middle-) aged adults no effect of 12 weeks daily supplementation with 1 g omega-3 on LPS-induced concentrations of IL-1β and TNF-α in PBMC was observed [26]. Results from human studies examining LPS-induced cytokine release or gene expression levels of inflammatory markers in response to omega-3 supplementation in old adults are therefore not consistent which in part might be due to the presence and type of underlying disease. Therefore, the clinical importance of our observations on the ageing immune system is uncertain, but it is likely that the additional supplementation with omega-3 fatty acids in moderate amounts caused no or only little effects on the immune response.

### 3.5. Sex-Related Differences

Since it has been recommended to consider the impact of sex on inflammation even in higher age [17], we performed sex-stratified analyses and compared changes between women and men. Sex-stratified analyses showed significant changes on various inflammatory markers in either women or men. However, only changes in circulating IL-10 and CCL-2 concentrations (Figure S1), gene expression levels of *IL1B* (Figure S2)*,* as well as the LPS-induced release of IL-1β and CCL-2, were significantly different between women and men (Figure S3). Progressive, unphysiological enlargement of adipocytes leads to an oxygen undersupply, resulting in cell necrosis with infiltration of macrophages and local inflammation, which in turn will extend to systemic inflammation on the long-term [27]. Since (visceral) adipose tissue triggers a pro-inflammatory milieu [17], the different response might partially be explained by higher abdominal fat mass as indicated by a higher waist/height ratio in our male compared with female participants (Table S1).

### 3.6. Strength and Limitations

One strength of this trial is the variety of assessments of inflammatory markers in serum, on gene expression level as well as in response to LPS exposure, which allowed us to have a differentiated evaluation of the inflammatory response in old adults. Furthermore, adherence to the exercise and dietary interventions was high in both the protein and the protein + omega-3 group [16].

Limitations of our study are the small sample size and the relatively short intervention time. However, this analysis was a pilot trial using an explorative approach to determine combined effects of a dietary and exercise intervention on inflammation in community-dwelling old adults. The triple intervention of exercise, whey/protein enrichment and omega-3 supplementation, might impede the interpretation of the results, since all of them are associated with anti-inflammatory effects. However, we used a three-arm study design to distinguish the separate effects of the dietary interventions.

## 4. Materials and Methods

### 4.1. Study Design and Population Sample

This is a sub-analysis of an 8-week randomized controlled intervention trial in a 3-arm design, which has been described elsewhere in detail [16]. Briefly, we recruited community-dwelling old adults (65–85 years) without any malignant or severe disease, which are associated with chronic inflammation (e.g., rheumatism), diabetes mellitus type 1 and 2, dementia, or severe food allergies. The study was approved by the University of Potsdam ethics committee, registered at the German study register (DRKS00018995) and carried out in accordance with the Declaration of Helsinki. All participants gave written informed consent. Participants were instructed to refrain from alcohol and vigorous exercise the day prior to examination. Following an overnight fast, all measurements were performed at the German Institute of Human Nutrition Potsdam-Rehbruecke.

### 4.2. Anthropometric Measurements

Weight (kg), height (cm), and waist circumference (cm) were measured to subsequently calculate BMI (kg/m^2^) and waist/height ratio. Body composition expressed as FMI (kg/m^2^) was estimated with single frequency bioimpedance analysis (Bioimpedance Analyzer Quantum/S Akern, Florence, Italy) at 50 kHz with the participants lying in the supine position.

### 4.3. Dietary Assessment

Daily dietary intake was recorded using 3-day dietary protocols and calculated with the nutrition software EBISpro version 2016 (Dr. J. Erhart, Willstätt-Legelshurst, Germany).

### 4.4. Laboratory Assessments

All blood samples were collected between 8 and 10 a.m. after an overnight fast and stored at −80 °C until analysis. Commercial enzyme-linked immunosorbent assays (ELISA) were used to measure serum concentrations (pg/mL) of IL-6, IL-10, IL-1RA, CCL-2 (all BioVendor, Brno, Czech Republic), and HMGB-1 (ng/mL) (IBL International GmbH, Hamburg, Germany) according to manufacturer’s instructions. IL-6 to IL-10 was also expressed as ratio, reflecting cytokine balance. The omega-3 plasma index was measured in phospholipid fractions by gas chromatography as described elsewhere [28] and represents the sum of eicosapentaenoic acid (EPA) and docosahexaenoic acid (DHA) as percentage of the total fatty acid spectrum.

### 4.5. PBMC Isolation, RNA Extraction, and Gene Expression with qPCR

To isolate PBMC, blood was collected with 8 mL BD Vacutainer^®^ CPT™-tubes (Becton, Dickinson and Company, Franklin Lakes/NJ, USA) and centrifuged (1650× *g*, 20 min, 20 °C) within a two-hour window. After centrifugation, the PBMC layer was washed twice with phosphate-buffered saline and centrifuged (250× *g*, 15 min, 20 °C). Isolated cells were counted and stored at −80 °C until further analyses.

Ribonucleic acid (RNA) was extracted from isolated PBMC using the NucleoSpin^®^ RNA Plus kit (Macherey-Nagel, Düren, Germany). Synthesis of cDNA was performed with High-Capacity cDNA Reverse Transcription Kit (Applied Biosystems™, Waltham/MA, USA). Gene expression was assessed by the quantitative real-time PCR (qPCR) using the SYBR™ Green dye (Power SYBR™ Green PCR Master Mix, Applied Biosystems™, Waltham/MA, USA) and specific primers (Table S2). All samples were measured as triplicates in optical 384-well plates (Applied Biosystems™, Waltham/MA, USA) and quantified using the standard curve method (Applied Biosystems™, Waltham/MA, USA). Relative expression levels of the target genes IL-6 (*IL6*), IL-10 (*IL10*), IL-1RA (*IL1RN*), IL-1β (*IL1B*), CCL-2 (*CCL2*), and TNF-α (*TNFA*) were normalized to the housekeeping gene beta-2-microglobulin (*B2M*).

### 4.6. Ex Vivo Whole-Blood LPS Stimulation

Ex vivo stimulation of whole-blood samples with LPS was performed to evaluate immune cell capacity as described previously [29]. In brief, after the collection, heparinized whole-blood was immediately diluted in a 1:5 ratio with RPMI 1640 medium (Invitrogen/Life Technology, Carlsbad/CA, USA). Blood cultures (2 mL/well) were either stimulated with 100 ng LPS/mL (LPS from *Escherichia coli*, O55:B5, Sigma Aldrich, Taufkirchen, Germany) or the same amount of medium (as a control) and incubated in sterile TC dish 35 standard (Sarstedt AG & Co. KG, Nümbrecht, Germany) for 4 h, at 37 °C in 5% CO_2_. After incubation, samples were centrifuged (2000× *g*, 3 min, 20 °C) to remove cellular components and supernatants were frozen at −80 °C until further processing. LPS-induced concentrations (pg/mL) of IL-6, IL-10, IL-1RA, IL-1β, CCL-2, and TNF-α were measured with commercial ELISA (all BioVendor, Brno, Czech Republic).

### 4.7. Whole-Body Vibration Training and Home-Based Resistance Exercise

All participants trained once per week under guidance at the institute on a Galileo^®^ side-alternating vibration plate (Novotec Medical GmbH, Pforzheim, Germany) and performed three times weekly a home-based and age-appropriate body weight exercise program, which consisted of seated crunches, marching, squats, chair rises, and chair dips (approx. 45 min). To account for individual physical conditions and accordingly avoid under- or overtraining, the training protocol for each participant started at their individual upper performance limit. To ensure progression, training protocols followed a weekly increase in vibration frequency (+2 Hz) and number of repetitions (+2). Adherence to the protocol was documented with training diaries.

### 4.8. Dietary Intervention

Participants were randomly assigned to either control, high-protein (protein) or high-protein, omega-3-enriched (protein + omega-3) diet groups. While the control group continued their usual diet, both protein-enriched groups received a high-protein diet (1.2–1.5 g protein/kg body weight/day), supported with a daily 300 mL whey drink (27 g protein; including 4 g leucine). In addition, the protein + omega-3 group was supplemented daily with 3.5 mL algae oil (2195 mg omega-3 fatty acids; including 1397 mg DHA, 749 mg EPA, and 49 mg docosapentaenoic acid). To evaluate compliance, left-overs of the whey and omega-3 supplements were weighed back at the end of the study, and additionally, omega-3 index was measured in plasma.

### 4.9. Data Analysis

Statistical analyses were performed with SPSS Statistics version 25 (IBM Corp., Chicago/IL, USA). Data distribution was checked using Kolmogorov–Smirnov tests and presented as either mean ± standard deviation or median with interquartile range (IQR). Extreme outliers of relative changes after treatment, identified with boxplots and defined as cases lying 3*IQR distant, were removed from the data set. Within-group comparisons before and after treatment were tested with either paired *t*-test or Wilcoxon rank test.

Generalized linear mixed models with random effect on subjects were used to investigate group × time-interaction effects between groups and presented as estimated mean with 95% confidence interval (95% CI). All models were adjusted for age, sex, and FMI, since we observed significant associations with our markers of interest. Inflammatory markers were also adjusted for baseline values, when significantly different between groups at baseline. Due to skewness, inflammatory markers have been log-transformed before analyses. In addition, we performed sex-stratified analyses with mixed models adjusted for age and FMI, and compared group-specific changes between sexes with either unpaired *t*-test or Mann–Whitney U-test. Statistical significance was assumed at *p* < 0.05.

## 5. Conclusions

Eight weeks of a high-protein, omega-3-enriched diet combined with exercise decreased circulating anti-inflammatory markers IL-10 and IL-1RA. In men, the pro-inflammatory markers IL-6, CCL-2, and HMGB-1 were reduced following a high-protein, omega-3-supplemented diet. A high-protein diet attenuated anti-inflammatory IL-1RA on gene expression levels in PBMC. Exercise alone resulted in a lower CCL-2 response to ex vivo LPS exposure in whole-blood-cultures, which is likely attributable to the effects seen in men.

## Figures and Tables

**Table 1:** Baseline characteristics and inflammatory markers, displayed for control, high-protein (protein), and high-protein, omega-3 enriched (protein + omega-3) groups.

	Control (*n* = 20)	Protein (*n* = 20)	Protein + Omega-3 (*n* = 21)
Sex [female/male]	10/10	11/9	11/10
Age [years]	69.9 ± 4.5	71.5 ± 4.6	70.4 ± 5.1
Medication [n]	3 ± 2	2 ± 2	2 ± 2
Waist/height ratio	0.58 ± 0.06	0.60 ± 0.05	0.59 ± 0.06
Body mass index [kg/m^2^]	26.9 ± 2.7	28.2 ± 2.3	27.8 ± 2.7
Fat mass index [kg/m^2^]	8.75 ± 2.38	9.44 ± 2.28	9.37 ± 1.98
Serum concentrations			
IL-6 (pg/mL)	2.97 (1.34)	2.62 (1.65)	3.01 (2.10)
IL-10 (pg/mL)	8.21 (4.28)	7.84 (3.55)	9.43 (4.21)
IL-6/IL-10 ratio	0.37 (0.38)	0.34 (0.25)	0.34 (0.23)
IL-1RA (pg/mL)	923 ± 366	790 ± 432	1228 ± 661 ^a^
CCL-2 (pg/mL)	214 (119)	229 (118)	251 (62)
HMGB-1 (ng/mL)	0.38 (1.29)	0.29 (0.50)	0.25 (0.64)
Gene expression levels in PBMC			
*IL6*	0.03 (0.04)	0.05 (0.07)	0.05 (0.05)
*IL10*	0.19 (0.20)	0.17 (0.28)	0.16 (0.21)
*IL1RN*	0.05 (0.03)	0.05 (0.01)	0.05 (0.01)
*IL1B*	0.04 (0.03)	0.04 (0.02)	0.04 (0.01)
*CCL2*	0.004 (0.007)	0.005 (0.005)	0.005 (0.003)
*TNFA*	0.56 (0.28)	0.66 (0.24)	0.57 (0.19)
LPS-induced concentrations in whole-blood cultures			
IL-6 (pg/mL)	7618 (3598)	6407 (5659)	7059 (5004)
IL-10 (pg/mL)	2.48 (7.12)	3.41 (4.32)	2.66 (2.82)
IL-1RA (pg/mL)	6194 (2640)	5116 (4605)	4913 (4310)
IL-1β (pg/mL)	850 (587)	954 (660)	879 (440)
CCL-2 (pg/mL)	122 (100)	109 (131)	101 (82)
TNF-α (pg/mL)	3245 ± 1488	2787 ± 1394	3117 ± 1151

Continuous variables are expressed as mean ± standard deviation or median (interquartile range). ^a^ Significantly different to protein group. CCL-2/*CCL2* c-c motif chemokine ligand-2; IL interleukin, β/*B* beta, RA/*RN* receptor antagonist; HMGB-1 high-mobility group box-1; LPS lipopolysaccharide; PBMC peripheral blood mononuclear cell; TNF- α/*TNFA* tumor necrosis factor-α.

## Data Availability

The data that support the findings of this study are available from the corresponding author upon reasonable request.

## Supplementary Materials

The following supporting information can be downloaded at: https://www.mdpi.com/article/10.3390/ijms24020928/s1.

## Abbreviations

95% CI	95% confidence interval
β/*B*	beta
*B2M*	beta-2-microglobulin
BMI	body mass index
CCL-2/*CCL2*	c-c motif chemokine ligand-2
DHA	docosahexaenoic acid
ELISA	enzyme-linked immunosorbent assays
EPA	eicosapentaenoic acid
FMI	fat mass index
HMGB-1	high-mobility group box-1
IL	interleukin
IQR	interquartile range
LPS	lipopolysaccharide
PBMC	peripheral blood mononuclear cells
qPCR	quantitative real-time polymerase chain reaction
RA/*RN*	receptor antagonist
RNA	ribonucleic acid
TNF-α/*TNFA*	tumor necrosis factor-α
