# Supplementary material for: Effects of Exercise and Omega-3-Supplemented, High-Protein Diet on Inflammatory Markers in Serum, on Gene Expression Levels in PBMC, and after Ex Vivo Whole-Blood LPS Stimulation in Old Adults
Source: Int J Mol Sci. 2023 Jan 4;24(2):928. doi: 10.3390/ijms24020928 (PMC9863449; doi:10.3390/ijms24020928)
Supplement: Supplementary file 1 [file ijms-24-00928-s001.zip › ijms-2100277-supplementary.pdf]

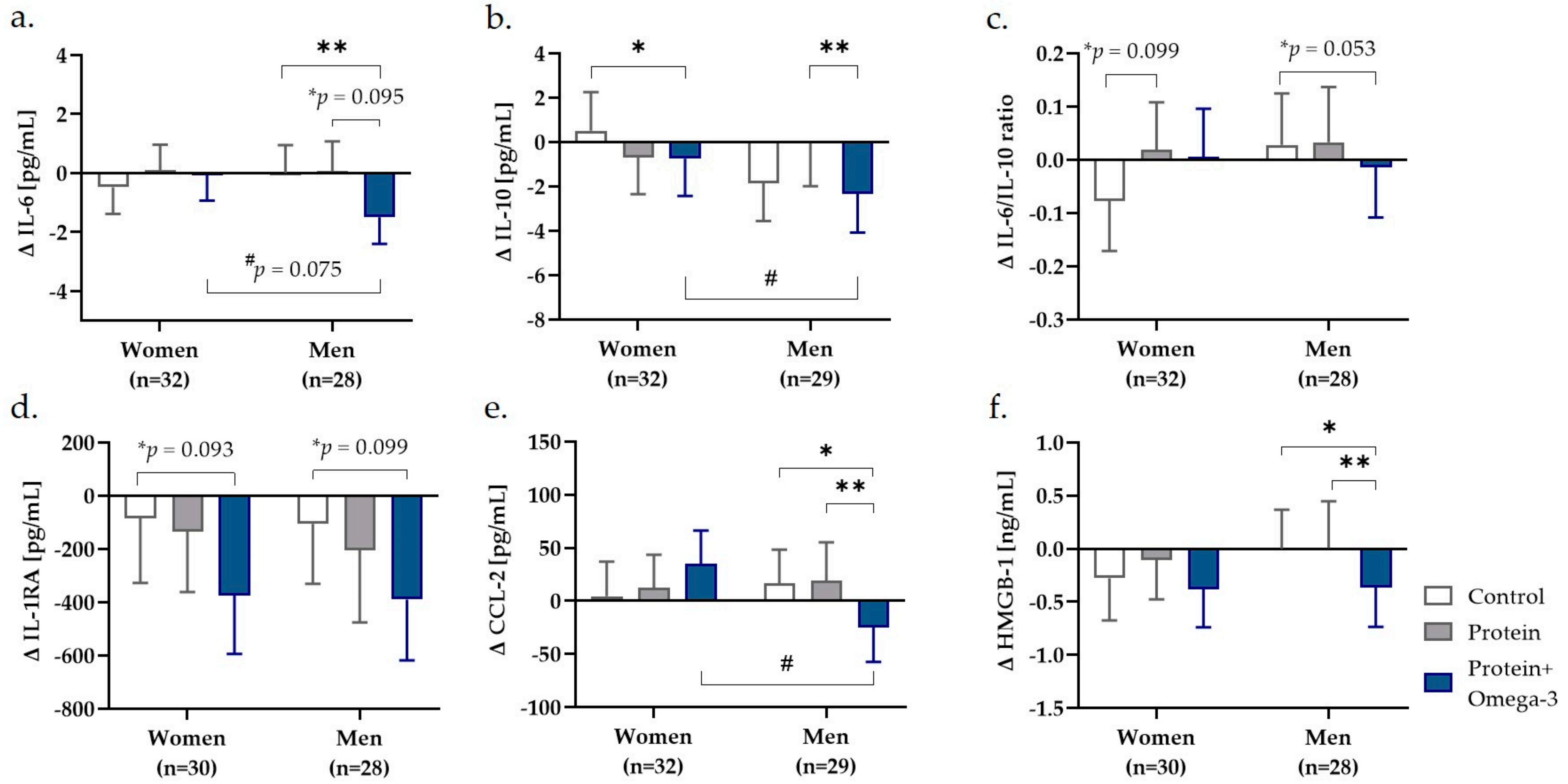

Figure S1. Delta (Δ) of serum concentrations of (**a**) IL-6, (**b**) IL-10, (**c**) IL-6/IL-10 ratio, (**d**) IL-1RA, (**e**) CCL-2, and (**f**) HMGB-1 presented as estimated mean with 95% confidence interval after eight weeks of control, high-protein (protein) or high-protein, omega-3-enriched (protein + omega-3) diet, displayed separately for women and men. Please note: Changes are shown as absolute values, but have been log-transformed before analyses. * $p < 0.05$, ** $p < 0.01$ obtained from generalized linear mixed models with random effects on subjects, adjusted for age and fat mass index to compare intervention effects within sex. # $p < 0.05$ obtained from comparisons of group-specific changes between sex with either unpaired t-test or Mann-Whitney U test. CCL-2 c-c motif chemokine ligand-2; HMGB-1 high-mobility group box-1; IL interleukin, RA receptor antagonist.

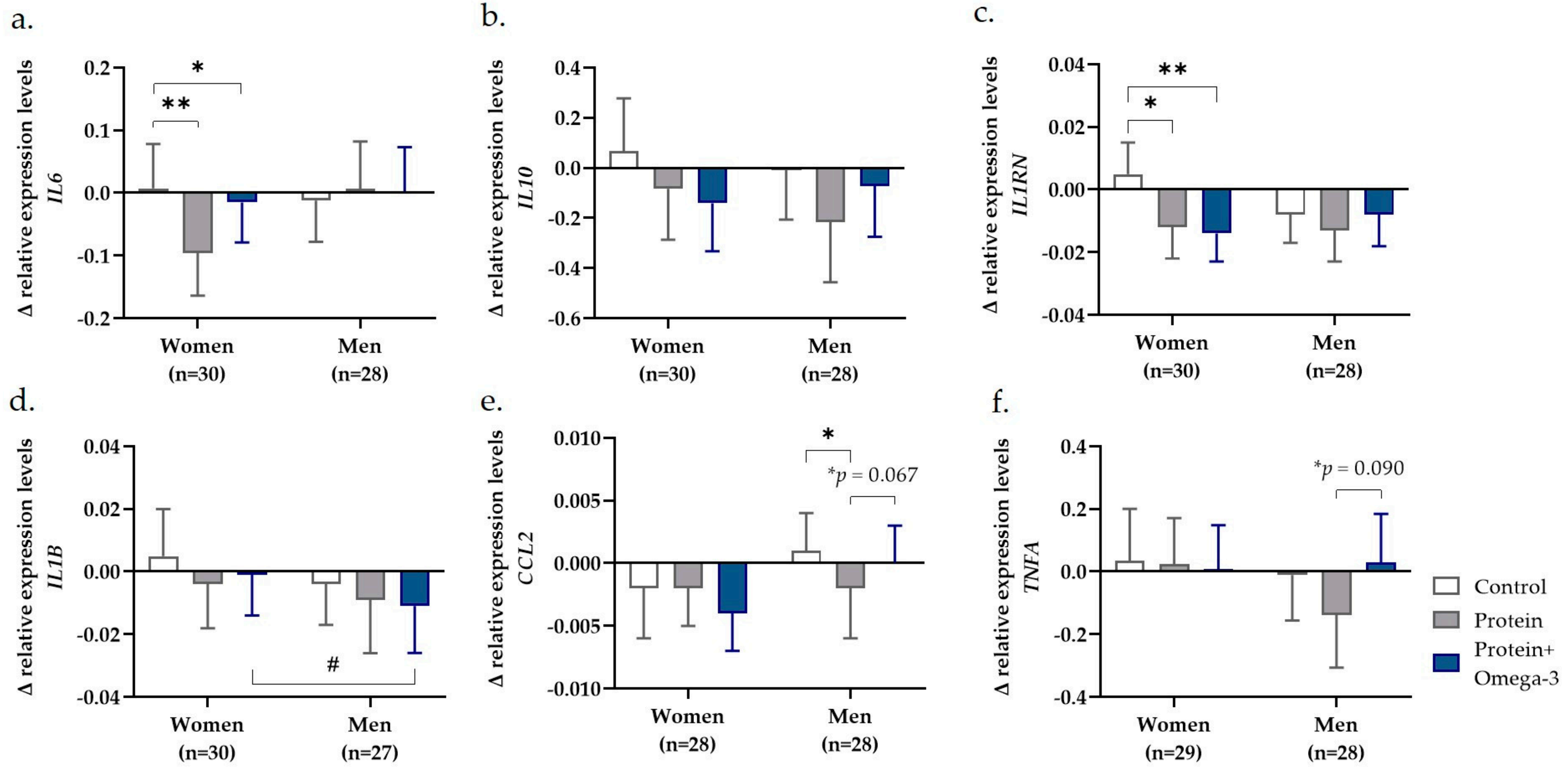

Figure S2. Delta (Δ) of gene expression levels of (**a**) *IL6*, (**b**) *IL10*, (**c**) *IL1RN*, (**d**) *IL1B*, (**e**) *CCL2*, and (**f**) *TNFA* presented as estimated mean with 95% confidence interval after eight weeks of control, high-protein (protein) or high-protein, omega-3-enriched (protein + omega-3) diet, displayed separately for women and men. Please note: Changes are shown as absolute values, but have been log-transformed before analyses. * $p < 0.05$, ** $p < 0.01$ obtained from generalized linear mixed models with random effects on subjects, adjusted for age and fat mass index to compare intervention effects within sex. # $p < 0.05$ obtained from comparisons of group-specific changes between sex with either unpaired t-test or Mann-Whitney U test. *CCL2* c-c motif chemokine ligand-2; *IL* interleukin, *B* beta, *RN* receptor antagonist; *TNFA* tumor necrosis factor-α.

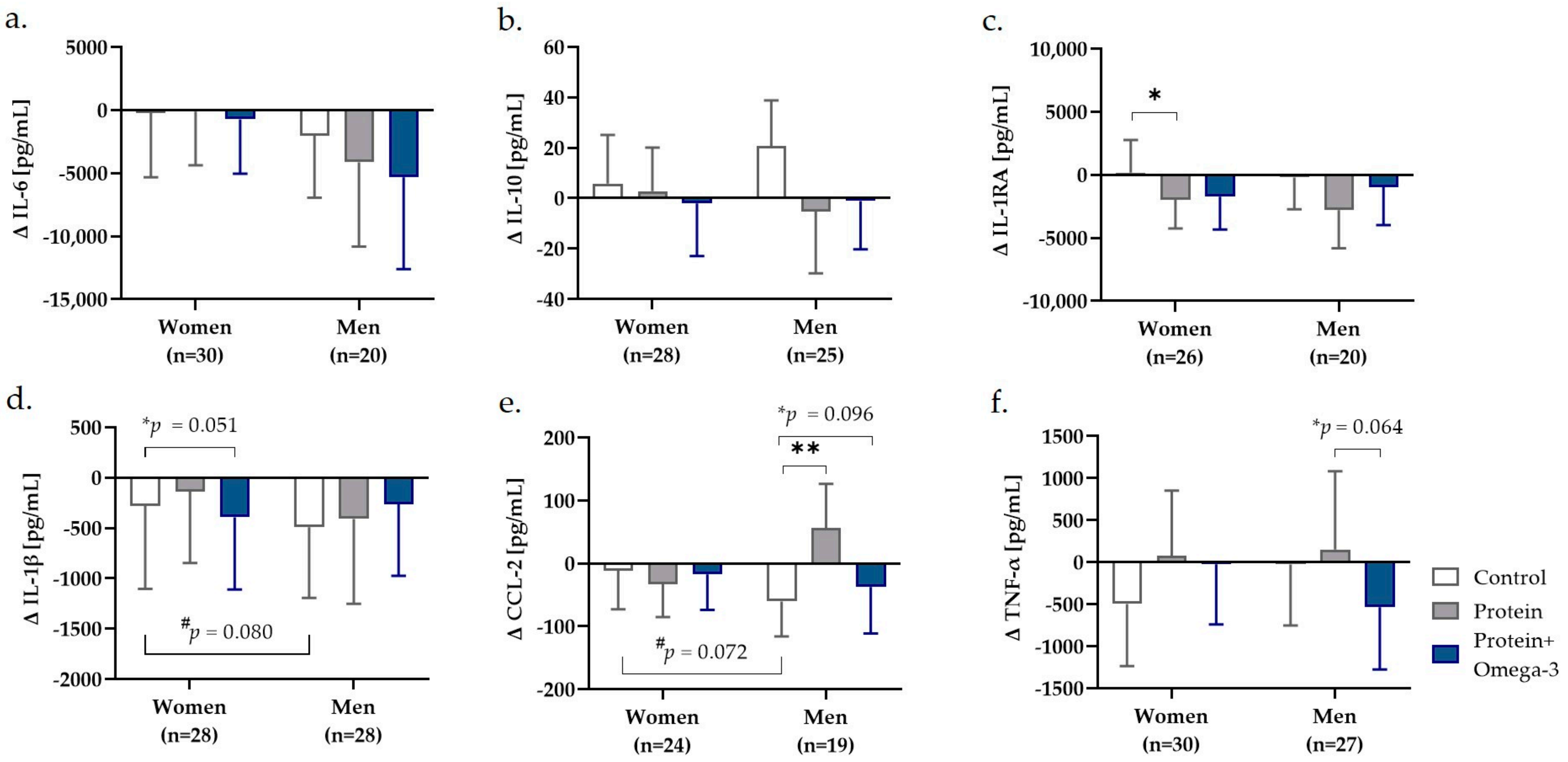

Figure S3. Delta (Δ) of ex vivo whole-blood LPS-stimulated concentrations of (**a**) IL-6, (**b**) IL-10, (**c**) IL-1RA, (**d**) IL-1β, (**e**) CCL-2, and (**f**) TNF-α presented as estimated mean with 95% confidence interval after eight weeks of control, high-protein (protein) or high-protein, omega-3-enriched (protein + omega-3) diet, displayed separately for women and men. Please note: Changes are shown as absolute values, but were log-transformed before analyses. * $p < 0.05$, ** $p < 0.01$ obtained from generalized linear mixed models with random effects on subjects, adjusted for age and fat mass index to compare intervention effects within sex. # p obtained from comparisons of group-specific changes between sex with either unpaired t-test or Mann-Whitney U test. CCL-2 c-c motif chemokine ligand-2; IL interleukin, RA receptor antagonist; TNF-α tumor necrosis factor-α.

Table S1. Key characteristics compared between women and men at baseline.

	**Women (*n*=32)**	**Men (*n*=29)**	***p*-value**
Age [years]	68.0 ± 2.2	72.9 ± 5.9	0.003
Waist/height ratio	0.58 ± 0.05	0.61 ± 0.06	0.064
Body mass index [kg/m²]	26.8 ± 2.3	28.1 ± 2.8	0.033
Fat mass index [kg/m²]	10.1 ± 1.7	7.7 ± 2.1	<0.001
Serum concentrations			
IL-6 (pg/mL)	2.39 (1.07)	3.19 (1.93)	0.011
IL-10 (pg/mL)	7.75 (3.14)	9.43 (4.89)	0.038
IL-6/IL-10 ratio	0.37 ± 0.21	0.41 ± 0.23	0.474
IL-1RA (pg/mL)	868 ± 440	985 ± 432	0.428
HMGB-1 (ng/mL)	0.23 (0.77)	0.36 (1.28)	0.492
CCL-2 (pg/mL)	220 ± 75	232 ± 78	0.162
Gene expression levels in PBMC			
IL6	0.046 (0.048)	0.033 (0.046)	0.182
IL10	0.142 (0.155)	0.243 (0.224)	0.168
IL1RN	0.048 ± 0.011	0.052 ± 0.015	0.647
IL1B	0.035 (0.012)	0.042 (0.018)	0.214
CCL2	0.006 (0.004)	0.005 (0.004)	0.384
TNFA	0.614 ± 0.207	0.642 ± 0.237	0.811
LPS-induced concentrations in whole-blood cultures			
IL-6 (pg/mL)	6002 (3862)	12,060 (3200)	0.010
IL-10 (pg/mL)	4.08 ± 2.72	3.37 ± 2.38	0.573
IL-1RA (pg/mL)	5168 (4275)	6451 (3513)	0.249
IL-1β (pg/mL)	761 ± 262	1113 ± 387	0.008
CCL-2 (pg/mL)	96 (70)	153 (93)	0.225
TNF-α (pg/mL)	2333 ± 1020	3583 ± 1024	<0.001

Continuous variables are expressed as mean ± standard deviation or median (interquartile range). *p*-value obtained from either t-test or Mann-Whitney U test. CCL-2/*CCL2* c-c motif chemokine ligand-2; IL interleukin, β/*B* beta, RA/*RN* receptor antagonist; HMGB-1 high-mobility group box-1; LPS lipopolysaccharide; PBMC peripheral blood mononuclear cell; TNF-α/*TNFA* tumor necrosis factor-α.

Table S2. Primers used for gene expression analysis by qPCR.

Gene name		Primer sequence (5` → 3`)
B2M	forward	CTA TCC AGC GTA CTC CAA AG
	reverse	AAA CCC AGA CAC ATA GCA AT
CCL2	forward	CAT AGC AGC CAC CTT CAT TCC
	reverse	TCT GCA CTG AGA TCT TCC TAT TGG
IL6	forward	AGC CCT GAG AAA GGA GAC ATG TA
	reverse	TCT GCC AGT GCC TCT TTG CT
IL10	forward	ACG GCG CTG TCA TCG ATT
	reverse	GGC ATT CTT CAC CTG CTC CA
IL1B	forward	GCA ATG AGG ATG ACT TGT TCT TTG
	reverse	CAG AGG TCC AGG TCC TGG AA
IL1RN	forward	ACC TCC CTC ATG GAC TGG TCT T
	reverse	CTT CCT CCC TCA TTC CAC CTT C
TNFA	forward	GGA CCT CTC TCT AAT CAG CCC TC
	reverse	TCG AGA AGA TGA TCT GAC TGC C

B2M beta-2-microglobulin (housekeeping gene); *CCL2* c-c motif chemokine ligand-2; *IL* interleukin, *B* beta, *RN* receptor antagonist; *TNFA* tumor necrosis factor-α.
